# First Complete Cytochrome B Sequences and Molecular Taxonomy of Bat Species from Sri Lanka

**DOI:** 10.3390/ani12131674

**Published:** 2022-06-29

**Authors:** Thejanee Perera, Franziska Schwarz, Therese Muzeniek, Sahan Siriwardana, Beate Becker-Ziaja, Inoka C. Perera, Shiroma Handunnetti, Jagathpriya Weerasena, Gayani Premawansa, Sunil Premawansa, Andreas Nitsche, Wipula Yapa, Claudia Kohl

**Affiliations:** 1 Institute of Biochemistry, Molecular Biology and Biotechnology, University of Colombo, 00300 Colombo, Sri Lanka; shiromah@ibmbb.cmb.ac.lk (S.H.); jagath@ibmbb.cmb.ac.lk (J.W.); 2 IDEA (Identification of Emerging Agents) Laboratory, Department of Zoology and Environment Sciences, University of Colombo, 00300 Colombo, Sri Lanka; sahan@zoology.cmb.ac.lk (S.S.); icperera@sci.cmb.ac.lk (I.C.P.); suviprema@gmail.com (S.P.); wipula@gmail.com (W.Y.); 3 Robert Koch Institute, Centre for Biological Threats and Special Pathogens, Highly Pathogenic Viruses (ZBS 1), 13353 Berlin, Germany; schwarzf@rki.de (F.S.); muzeniekt@rki.de (T.M.); nitschea@rki.de (A.N.); kohlc@rki.de (C.K.); 4 Centre for International Health Protection, Public Health Laboratory Support (ZIG 4), Robert Koch Institute, 13353 Berlin, Germany; beate.becker-ziaja@bnitm.de; 5 Colombo North Teaching Hospital, 11010 Ragama, Sri Lanka; gavisprema@gmail.com

**Keywords:** molecular identification of bats, bat phylogeny, Sri Lankan bats, molecular phylogeny, One Health, molecular taxonomy, cryptic species

## Abstract

**Simple Summary:**

The aim of our study was to address the research gap in the molecular taxonomy of Sri Lankan bats. The accurate identification of animals plays a major role in observing them in their natural environments and hence understanding possible disease-transmitting pathways from animals to humans. Being a tropical country, Sri Lanka has a high density of animals. There are 30 different species of bats described in Sri Lanka. Until now, the animals have been identified by observing their physical features. However, the visual identification of animals is not accurate because closely related animal groups may show similar physical features. During our study, we accurately differentiated five bat groups living in one of the largest caves in Sri Lanka by using a more sophisticated laboratory technique. Using molecular techniques, we were able to provide more accurate results than by the visual identification of the bats. The results from our study are stored in the NCBI database as a baseline for a repository of Sri Lankan bats. With the new sequence data provided here, we filled the gap concerning the molecular taxonomy of bat species of the entire region and we contributed to the future conservation and systematic studies of these mammals.

**Abstract:**

This is the first report on the molecular identification and phylogeny of the *Rousettus leschenaultii* Desmarest, 1810, *Rhinolophus rouxii* Temminck, 1835, *Hipposideros speoris* Schneider, 1800, *Hipposideros lankadiva* Kelaart, 1850, and *Miniopterus fuliginosus* Kuhl, 1817, bat species in Sri Lanka, inferred from analyses by mitochondrially encoded cytochrome b gene sequences. Recent research has indicated that bats show enormous cryptic genetic diversity. Moreover, even within the same species, the acoustic properties of echolocation calls and morphological features such as fur color could vary in different populations. Therefore, we have used molecular taxonomy for the accurate identification of five bat species recorded in one of the largest cave populations in Sri Lanka. The bats were caught using a hand net, and saliva samples were collected non-invasively from each bat by using a sterile oral swab. Nucleic acids were extracted from the oral swab samples, and mitochondrial DNA was amplified by using primers targeting the mitochondrially encoded cytochrome b gene. This study reports the first molecular evidence for the identification of five bat species in Sri Lanka. Our findings will contribute to future conservation and systematic studies of bats in Sri Lanka. This study will also provide the basis for a genetic database of Sri Lankan bats which will contribute significantly to the investigation of potentially zoonotic bat viruses.

## 1. Introduction

Bats (order: Chiroptera) are the second largest group of mammals documented with 1293 species in the world [1]. Being a tropical country, with long day lengths and seasonal rainfall, Sri Lanka is endowed with high biological diversity. So far, 30 species of bats have been recorded in Sri Lanka [2]. Consequently, bats are the largest group of mammals recorded in Sri Lanka, accounting for approximately 1/3 of all mammalian species present on the island [2]. 

All the Megachiropterans and the members of the superfamily Rhinolopoidea are grouped in the suborder Yinpterochiroptera, and the remaining microchiropteran species are included in the suborder Yangochiroptera [3,4]. Taxonomic trees and phylogenetic reconstructions are based on the genetic information of animals. Housekeeping genes have low evolutionary rates and are therefore often suitable for accurate species identification and molecular taxonomy [5]. These genes show low intraspecific variation and high interspecific variation. The mitochondrially encoded cytochrome b (cytb) gene is a commonly used housekeeping gene that is involved in oxidative phosphorylation in cellular respiration. Whole gene sequences of the cytb can be found in the National Center for Biotechnology Information (NCBI) database.

Although some studies have been carried out on ecological and behavioral aspects of Sri Lankan bats, research on molecular identification is limited to a single genus *Cynopterus* [6]. Wellawaya Wavulgalge cave in Sri Lanka houses one of the largest sympatric colonies found on the island, occupied by five species of bats forming a unique colony [7]. Approximately 100,000 individual bats inhabit the cave. Based on external morphometric characteristics, these five species were characterized as *Rousettus leschenaultii* Desmarest, 1810, *Rhinolophus rouxii* Temminck, 1835, belonging to suborder Yinpterochiroptera, and *Hipposideros speoris* Schneider, 1800, *Hipposideros lankadiva* Kelaart, 1850, and *Miniopterus fuliginosus* Kuhl, 1817, belonging to suborder Yangochiroptera. In this study, amplification of the cytb gene was utilized for the accurate identification of these five bat species inhabiting the Wavulgalge cave.

## 2. Materials and Methods

Investigative research on Sri Lankan bats was approved by the Department of Wildlife Conservation, Sri Lanka (permit No. WL/3/2/05/18) and conducted in accordance with the Fauna and Flora Protection Ordinance (FFPO) of Sri Lanka. Ethical clearance for the study was obtained from the Institute of Biology, Sri Lanka (ERC IOBSL 170 01 18). Samples were collected in a non-invasive manner. Animal capturing, handling, and sampling was done in accordance with the guidelines of the American Society of Mammalogists for the use of wild mammals in research and education [8]. The sampling site is a natural cave situated in Nikapitiya, Wellawaya (6°43′00.0″ N 81°03′00.0″ E) in the Monaragala district, Sri Lanka. The roost is known as Wavulgalge cave. It is a lithophilic type of roost and one of the largest sympatric bat roosts found on the island. This underground cave covers over 45,000 m^2^ of area. The Wavulgalge cave provides a stable microclimate and protection from predators and extreme weather conditions [7]. Sample collection was carried out during March and July of 2018 and in January of 2019.

Personal protective equipment (PPE) such as safety gloves, safety glasses, and FFP3 masks were used during animal capturing, handling, and sample collection to reduce the potential risk of zoonotic or anthropozoonotic pathogen transmission.

A total of 74 bats were captured by using hand nets while they were emerging from the roost in the evenings. Captured live bats were placed into cotton bags and kept in ambient temperature until further processing. Bat species were macroscopically identified by using external morphological features such as size and shape of nose. Morphometric parameters and locational data were recorded in the data sheets. Bats were released at the cave entrance immediately after recording the morphometric data and the collection of samples. Saliva samples were collected from each bat by using a sterile oral swab and snap-frozen by using liquid nitrogen. Collected samples were transported to the laboratory in a dry-shipper and stored at −80 °C until further analysis. Laboratory analyses of the samples were carried out under BSL-2 conditions at the Robert Koch Institute, Berlin, Germany.

Phosphate-buffered saline (200 µL) was added into each vial containing a dry oral swab and vortexed vigorously. QIAamp Viral RNA Mini kit (Qiagen, Hilden, Germany) was used to extract the nucleic acids from oral swab samples according to the manufacturer’s instructions. Full cytb gene was amplified using RrFP and RrRP primers as previously described [9]. PCR products were purified by using MSB Spin PCRapace purification kit protocol (Invisorb, Invitek GmbH, Berlin, Germany) following the manufacturer’s instructions. Purified products were sequenced on the Applied Biosystems 3500 Dx Genetic Analyzer by using the corresponding forward and reverse primers for each strand. The BigDye Terminator v3.1 Cycle Sequencing Kit (Thermo Fisher Scientific, Waltham, MA, USA) was used to perform Sanger sequencing reactions.

The sequences obtained were analyzed using Geneious Prime 2020.0.5 software. Primers were trimmed from the sequences and assembled to consensus sequences. The consensus sequence of each bat was compared with the sequences in the NCBI database by using the basic local alignment search tool (BLAST) to identify the respective species and calculate the statistical significance of the matches. The results were compared with the initial morphological identification.

Further data analyses were carried out by using MEGA X: Molecular Evolutionary Genetics Analysis across computing platforms (Kumar, Stecher, Li, Knyaz, and Tamura 2018). Cytb gene sequences (1140 bp) of reference bat species were downloaded from the NCBI database and aligned with the consensus sequences obtained from this study by using the MUSCLE alignment tool in the MEGA X application. The best substitutional model for the alignment was calculated by using the J model test [10]. Phylogenetic reconstruction was carried out by using the MrBayes v3.2.6 (Huelsenbeck, J. P. and F. Ronquist. 2001) Markov chain Monte Carlo (MCMC) method (parameters were as follows: substitution model, GTR, rate variation, equal; chain length 10 million; burn-in, 30%; sampling frequency, 200; *Felis catus* was used as an outgroup) [11]. Phylogenetic trees were visualized by using FigTree software (http://tree.bio.ed.ac.uk/software/figtree/ (accessed on 10 May 2021)) and posterior probabilities were calculated for each node [11]. A heat map was calculated based on the nucleotide alignment and percentage of identity.

## 3. Results

The obtained sequences are the first mitochondrially encoded cytochrome b full gene sequence data of Sri Lankan bats. A total of 74 full cytb sequences of 1140 bp in length were obtained for *Rousettus leschenaultii*, *Rhinolophus rouxii, Hipposideros speoris, Hipposideros lankadiva*, and *Miniopterus fuliginosus*. Table S1 in the supplementary material provides all data collected for the 74 individual bats.

**Figure 1:**
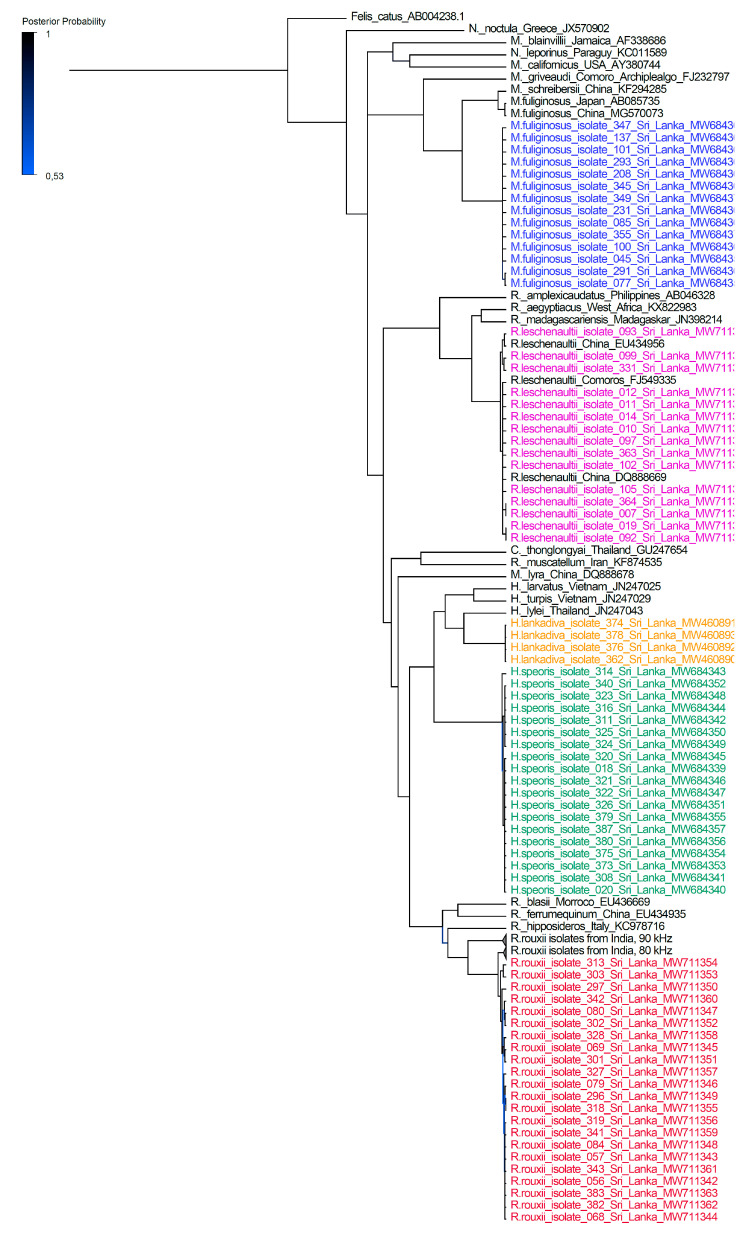
Phylogenetic reconstruction of bat species based on the full mitochondrially encoded cytochrome b gene (1140 bp) in comparison to other species of bats. The posterior probability is displayed as gradient shade of the tree lines ranging from 0.53 to 1.0. Branches of *R. rouxii* from India (90 kHz and 80 kHz) are collapsed to increase visibility. Reconstruction was calculated by using the MrBayes MCMC method (parameters were as follows: substitution model, GTR, rate variation, equal; chain length 10 million; burn-in, 30%; sampling frequency, 200; *Felis catus* was used as an outgroup). Reconstructed trees were visualized by using FigTree and posterior probabilities were depicted for each node (http://tree.bio.ed.ac.uk/software/fgtree/ (accessed on 10 May 2021)).

Phylogenetic trees were constructed using molecular data and used to make inferences to understand the evolutionary relationship among taxa. Phylogenetic reconstruction allocated the sequences of the five Sri Lankan bat species to the corresponding bat families within the species tree (Figure 1).

*Miniopterus fuliginosus* and *H. lankadiva* showed very low levels of intraspecies variation. Sequences from *H. speoris* appeared to be divided into two distinct sister-clades. Sequences from *Rousettus leschenaultii* were also divided into two clades while showing a close relationship to sequences obtained from *R. leschenaultii* from China. Sequences obtained from *R. rouxii* were clustered as a distinct sister-clade to *R. rouxii* sequences obtained from India. According to the phylogenetic tree, Sri Lankan *R. rouxii* were more closely related to the *R. rouxii* species identified in Srirangapattana and Moodbidri regions than to those identified in Yercaud and Sirumalai regions in India.

The relationships observed by phylogenetic reconstructions were further supported by the heat map of distances, based on the percentage of genetic identity for all five bat species (Figure S1, Figure S2, Figure S3 and Figure S4 supplementary Material).

## 4. Discussion

Bat species in Sri Lanka were first described by Kelaart in 1852 [12]. Later, a more detailed study of mammalian species in Sri Lanka was carried out based on their morphological features [13]. Species descriptions of the Sri Lankan mammals by Phillips are still used as the reference guide for the identification of bat species in Sri Lanka [14]. Many morphologically similar species have been recorded in neighboring South Asian countries [9]. With this study, we provide the first data for accurate molecular identification of five bat species inhabiting one of the largest sympatric bat colonies in Sri Lanka.

Furthermore, we provide the first phylogenetic tree for selected Sri Lankan bats based on cytb genealogy. Our data analysis further supports the recent classification of bats into Yinpterochiroptera and Yangochiroptera suborders [4]. The phylogenetic tree resulted in the monophyletic clustering of both Hipposideridae, Rhinolophidae, and Pteropodidae bats and showed a clear divergence from the Vespertilionidae bats.

In contrast to other bat species inhabiting the cave, *M. fuliginosus* bats migrate to the Wavulgalge cave from nearby roosts during their maternity period, using the Wavulgalge cave as a maternity roost [15]. However, the sequence analysis data showed less variation between individual *M. fuliginosus* bats, suggesting that, based on the cytb gene sequences of *M. fuliginosus* bats from different locations in Sri Lanka, this species is highly conserved (Supplementary Figure S1 and Figure S2).

A total of four bats belonging to *H. lankadiva* were sampled during our field visits. Molecular data indicated that all four bats are highly similar to each other and more closely related to *Hipposideros lylei* (Thailand), *H. larvatus* (Vietnam), and *H. turpis* (Vietnam) bat species than *H. speoris* identified from Sri Lanka (Supplementary Figure S1).

The phylogenetic tree revealed that the *R. rouxii* species from the Wavulgalge cave were closely related to *R. rouxii* identified in the Karnataka district in India. There were two phonic types of *R. rouxii* recorded in India with echolocation call frequencies above 90 kHz and below 85 kHz [9,16]. According to the phylogenetic data analysis from our study, it was revealed that the Sri Lankan *R. rouxii* species was more closely related to the phonic type with echolocation call frequency below 85 kHz. Interestingly, the echolocation call frequencies of *R. rouxii* in Wavulgalge are below those of Rufous horseshoe bats recorded in India and fluctuate between 73.5 and 79 kHz [17].

*Rousettus leschenaultii* sequences obtained in this study appeared to be closely related to *R. leschenaultii* sequences from China (Supplementary Figure S1 and Figure S3). The phylogenetic reconstruction suggests that *R. leschenaultii* may have diverged from *R. aegyptiacus* and *R. madagascariensis* recorded in Africa. These three species are distinctly related to the *R. amplexicaudatus* recorded from the Philippines. This information is supported by a previous study carried out on *Rousettus* spp. phylogeny [18]. In our phylogenetic reconstruction *R. leschenaultii* appeared in two distinct clades of the population. *Rousettus leschenaultii* had the highest individual count for a single bat genus in Sri Lanka [2], was also the most common species recorded in the Wavulgalge cave, and occurs in all six bioclimatic zones of the island [14]. Therefore, more extensive research should be conducted to determine the genetic diversity of the *Rousettus* species in Sri Lanka.

It has been documented that zoonotic pathogens which have co-evolved in bats have a high risk of infecting other mammalian species, including humans [19]. Bats are found to carry a multitude of different microorganisms that include viruses, bacteria, fungi, and parasites [20,21]. Furthermore, the global interest of bats as potential reservoir hosts of zoonotic pathogens was highlighted recently as these flying mammals have been detected to be harboring coronaviruses [22]. In addition, recent studies revealed the presence of alpha- and betacoronaviruses in *Miniopterus fuliginosus* and *Rousettus leschenaultii* bats and paramyxoviruses in *Miniopterus fuliginosus* inhabiting the Wavulgalge cave, Sri Lanka [23,24,25]. Therefore, adequate PPE was worn during animal capturing, handling, and sample collection to reduce the potential risk of exposure to zoonotic or anthropozoonotic pathogens.

The prevalence of medically important pathogens in bats may be linked with the bat species, their geographical location, roosting nature, and other behavioral aspects [26,27,28]. Therefore, accurate species identification using molecular techniques will help predict the pathogen–host shifts and interspecies pathogen transmission in bats in the Wavulgalge cave in Sri Lanka in future studies [29,30].

## 5. Conclusions

Our study addressed the research gap in the molecular taxonomy of Sri Lankan bats. Our results were consistent with the species identification by morphological characteristics of the bat species in the Wavulgalge cave. Accurate identification of bats plays a critical role in conservation. Therefore, our findings will also contribute to future conservation and systematic studies of bats in Sri Lanka. Further data collected in our study will provide the basis for a genetic database of Sri Lankan bats working as reference data for future bat species identification in Sri Lanka.

## Data Availability

The data presented in this study is openly available in GenBank (https://www.ncbi.nlm.nih.gov/genbank/ accessed on 26 June 2022).

## Supplementary Materials

The following supporting information can be downloaded at: https://www.mdpi.com/article/10.3390/ani12131674/s1, Table S1: Overview on the 74 full MT-CYB sequences of 1140 bp collected for bats from the Wavulgalge cave, Sri Lanka; Figure S1: Heat maps based on the full MT-CYB gene (1140 bp) of *Hipposideros speoris*. Percentage of identity is depicted by color ranging from 70 percent (red) to 100 percent (blue); Figure S2: Heat maps based on the full MT-CYB gene (1140 bp) of *Miniopterus fuliginosus*. Percentage of identity is depicted by color ranging from 70 percent (red) to 100 percent (blue); Figure S3: Heat maps based on the full MT-CYB gene (1140 bp) of *Rousettus leschenaultii*. Percentage of identity is depicted by color ranging from 70 percent (red) to 100 percent (blue); Figure S4: Heat maps based on the full MT-CYB gene (1140 bp) of *Rhinolophus rouxii*. Percentage of identity is depicted by color ranging from 70 percent (red) to 100 percent (blue).
