# Supplementary material for: First Complete Cytochrome B Sequences and Molecular Taxonomy of Bat Species from Sri Lanka
Source: Animals (Basel). 2022 Jun 29;12(13):1674. doi: 10.3390/ani12131674 (PMC9264896; doi:10.3390/ani12131674)
Supplement: Supplementary file 1 [file animals-12-01674-s001.zip › animals-1714001-supplementary.pdf]

Supplementary File

First complete cytochrome b sequences and molecular taxonomy of bat species from Sri Lanka

Thejanee Perera[1, 3,*], Franziska Schwarz[2], Therese Muzeniek[2], Sahan Siriwardana[3], Beate Becker-Ziaja[4], Inoka C. Perera[3], Shiroma Handunnetti[1], Jagathpriya Weerasena[1], Gayani Premawansa[5], Sunil Premawansa[3], Andreas Nitsche[2], Wipula Yapa[3¶] and Claudia Kohl[2¶]

[1] Institute of Biochemistry, Molecular Biology and Biotechnology, University of Colombo, 00300 Colombo, Sri Lanka; thejanee90@gmail.com (T.P.); shiromah@ibmbb.cmb.ac.lk (S.H.); jagath@ibmbb.cmb.ac.lk (J.W.)
[2] Robert Koch Institute, Centre for Biological Threats and Special Pathogens, Highly Pathogenic Viruses (ZBS 1), 13353 Berlin, Germany; SchwarzF@rki.de (F.S.); muzeniekt@rki.de (T.M.); NitscheA@rki.de (A.N.); KohlC@rki.de (C.K.)
[3] IDEA (Identification of Emerging Agents) Laboratory, Department of Zoology and Environment Sciences, University of Colombo, 00300 Colombo, Sri Lanka; sahan@zoology.cmb.ac.lk (S.S.); suviprema@gmail.com (S.P.); icperera@sci.cmb.ac.lk (I.P.); wipula@gmail.com (W.Y.)
[4] Centre for International Health Protection, Public Health Laboratory Support (ZIG 4), Robert Koch Institute, 13353 Berlin, Germany; Becker-ZiajaB@rki.de (B.B.Z.)
[5] Colombo North Teaching Hospital, 11010 Ragama, Sri Lanka; gavisprema@gmail.com (G.P.)
* Correspondence: thejanee90@gmail.com; Tel.: +94 77 433 83 46

Table S1. Overview on the 74 full MT-CYB sequences of 1,140 bp collected for bats from Wavulgalge cave, Sri Lanka.

Bat identifier	Bat species (morphological)	Bat species (molecular)	% id with reference	Sex	Forearm length	Age	Sequence identifier	Accession number
056	*Rhinolophus rouxii*	*Rhinolophus rouxii*	98.53%	M	4.5		Seq056_RR	MW711342
057	*Rhinolophus rouxii*	*Rhinolophus rouxii*	98.53%	F	4.74	A	Seq057_RR	MW711343
068	*Rhinolophus rouxii*	*Rhinolophus rouxii*	98.53%	F	4.61	A	Seq068_RR	MW711344
069	*Rhinolophus rouxii*	*Rhinolophus rouxii*	98.32%	F	4.9		Seq069_RR	MW711345
079	*Rhinolophus rouxii*	*Rhinolophus rouxii*	98.74%	F	4.92		Seq079_RR	MW711346
080	*Rhinolophus rouxii*	*Rhinolophus rouxii*	98.64%	F	4.91	A	Seq080_RR	MW711347

Supplementary File

Bat identifier	Bat species (morphological)	Bat species (molecular)	% id with reference	Sex	Forearm length	Age	Sequence identifier	Accession number
084	*Rhinolophus rouxii*	*Rhinolophus rouxii*	98.43%	F	4.56	A	Seq084_RR	MW711348
296	*Rhinolophus rouxii*	*Rhinolophus rouxii*	98.64%	M	5.1	A	Seq296_RR	MW711349
297	*Rhinolophus rouxii*	*Rhinolophus rouxii*	99.06%	M	4.97	A	Seq297_RR	MW711350
301	*Rhinolophus rouxii*	*Rhinolophus rouxii*	98.53%	F	5.03	A	Seq301_RR	MW711351
302	*Rhinolophus rouxii*	*Rhinolophus rouxii*	98.74%	M		A	Seq302_RR	MW711352
303	*Rhinolophus rouxii*	*Rhinolophus rouxii*	99.16%	F	4.74	A	Seq303_RR	MW711353
313	*Rhinolophus rouxii*	*Rhinolophus rouxii*	98.74%	F	5.04	A	Seq313_RR	MW711354
318	*Rhinolophus rouxii*	*Rhinolophus rouxii*	98.64%	M	4.82	A	Seq318_RR	MW711355
319	*Rhinolophus rouxii*	*Rhinolophus rouxii*	98.43%	F	4.98	A	Seq319_RR	MW711356
327	*Rhinolophus rouxii*	*Rhinolophus rouxii*	98.53%		4.78	A	Seq327_RR	MW711357
328	*Rhinolophus rouxii*	*Rhinolophus rouxii*	98.74%	M	5.03	A	Seq328_RR	MW711358
341	*Rhinolophus rouxii*	*Rhinolophus rouxii*	98.43%	F	4.84	A	Seq341_RR	MW711359
342	*Rhinolophus rouxii*	*Rhinolophus rouxii*	98.64%	M	4.98	A	Seq342_RR	MW711360
343	*Rhinolophus rouxii*	*Rhinolophus rouxii*	98.53%	F	4.83	A	Seq343_RR	MW711361
382	*Rhinolophus rouxii*	*Rhinolophus rouxii*	98.53%	F	4.71	A	Seq382_RR	MW711362

Supplementary File

Bat identifier	Bat species (morphological)	Bat species (molecular)	% id with reference	Sex	Forearm length	Age	Sequence identifier	Accession number
383	*Rhinolophus rouxii*	*Rhinolophus rouxii*	98.53%	F	4.75	A	Seq383_RR	MW711363
007	*Rousettus leschenaultii*	*Rousettus*	99.79%				Seq007_RL	MW711364
010	*Rousettus leschenaultii*	*Rousettus*	100.00%				Seq010_RL	MW711365
011	*Rousettus leschenaultii*	*Rousettus*	99.79%				Seq011_RL	MW711366
012	*Rousettus leschenaultii*	*Rousettus*	99.79%				Seq012_RL	MW711367
014	*Rousettus leschenaultii*	*Rousettus*	100.00%				Seq014_RL	MW711368
019	*Rousettus leschenaultii*	*Rousettus*	99.79%				Seq019_RL	MW711369
092	*Rousettus leschenaultii*	*Rousettus*	99.79%	F	7.92	A	Seq092_RL	MW711370
093	*Rousettus leschenaultii*	*Rousettus*	100.00%	M			Seq093_RL	MW711371
097	*Rousettus leschenaultii*	*Rousettus*	100.00%	M	6.71	SA	Seq097_RL	MW711372
099	*Rousettus leschenaultii*	*Rousettus*	99.58%	M	7.83	A	Seq099_RL	MW711373
102	*Rousettus leschenaultii*	*Rousettus*	100.00%	F	6.66	SA	Seq102_RL	MW711374
105	*Rousettus leschenaultii*	*Rousettus*	99.90%	F	7.66	A	Seq105_RL	MW711375
331	*Rousettus leschenaultii*	*Rousettus*	100.00%	F	6.75	J	Seq331_RL	MW711376
363	*Rousettus leschenaultii*	*Rousettus*	100.00%	F	7.64	A	Seq363_RL	MW711377

Supplementary File

Bat identifier	Bat species (morphological)	Bat species (molecular)	% id with reference	Sex	Forearm length	Age	Sequence identifier	Accession number
364	*Rousettus leschenaultii*	*Rousettus*	99.79%	F	6.79	A	Seq364_RL	MW711378
018	*Hipposideros speoris*	*Hipposideros speoris*	91.42%				Seq018_HS	MW684339
020	*Hipposideros speoris*	*Hipposideros speoris*	91.42%				Seq020_HS	MW684340
308	*Hipposideros speoris*	*Hipposideros speoris*	91.42%	F	5.13	SA	Seq308_HS	MW684341
311	*Hipposideros speoris*	*Hipposideros speoris*	91.42%	F	5.04	SA	Seq311_HS	MW684342
314	*Hipposideros speoris*	*Hipposideros speoris*	91.42%	M	5.18	A	Seq314_HS	MW684343
316	*Hipposideros speoris*	*Hipposideros speoris*	91%	F	5.45	A	Seq316_HS	MW684344
320	*Hipposideros speoris*	*Hipposideros speoris*	91.42%	M	5.16	A	Seq320_HS	MW684345
321	*Hipposideros speoris*	*Hipposideros speoris*	91.42%	F	5.34	A	Seq321_HS	MW684346
322	*Hipposideros speoris*	*Hipposideros speoris*	91.42%	M	5.23	A	Seq322_HS	MW684347
323	*Hipposideros speoris*	*Hipposideros speoris*	91.42%	F	5.36	A	Seq323_HS	MW684348
324	*Hipposideros speoris*	*Hipposideros speoris*	91.42%	F	5.35	A	Seq324_HS	MW684349
325	*Hipposideros speoris*	*Hipposideros speoris*	91.42%	M	5.14	A	Seq325_HS	MW684350
326	*Hipposideros speoris*	*Hipposideros speoris*	91.42%	M	5.27	A	Seq326_HS	MW684351
340	*Hipposideros speoris*	*Hipposideros speoris*	91.42%	M	5.14	A	Seq340_HS	MW684352

Supplementary File

Bat identifier	Bat species (morphological)	Bat species (molecular)	% id with reference	Sex	Forearm length	Age	Sequence identifier	Accession number
373	*Hipposideros speoris*	*Hipposideros speoris*	91.42%	M	5.19	SA	Seq373_HS	MW684353
375	*Hipposideros speoris*	*Hipposideros speoris*	91.42%	F	5.34	SA	Seq375_HS	MW684354
379	*Hipposideros speoris*	*Hipposideros speoris*	91.42%	M	5.18	A	Seq379_HS	MW684355
380	*Hipposideros speoris*	*Hipposideros speoris*	91.42%	M	5.07	A	Seq380_HS	MW684356
387	*Hipposideros speoris*	*Hipposideros speoris*	91.42%	M	4.92	SA	Seq387_HS	MW684357
045	*Miniopterus*	*Miniopterus*	91.42%	M	4.3		Seq045_MF	MW684358
077	*Miniopterus*	*Miniopterus*	91.53%	F	4.61	A	Seq077_MF	MW684359
085	*Miniopterus*	*Miniopterus*	91.42%	M	4.76	A	Seq085_MF	MW684360
100	*Miniopterus*	*Miniopterus*	91.42%	M	4.5	A	Seq100_MF	MW684361
101	*Miniopterus*	*Miniopterus*	91.42%	F	4.67	A	Seq101_MF	MW684362
137	*Miniopterus*	*Miniopterus*	91.32%	F	4.7	A	Seq137_MF	MW684363
208	*Miniopterus*	*Miniopterus*	91.42%	F	4.68	A	Seq208_MF	MW684364
231	*Miniopterus*	*Miniopterus*	91.32%	M	4.76	A	Seq231_MF	MW684365
291	*Miniopterus*	*Miniopterus*	91.32%	M	4.78	A	Seq291_MF	MW684366
293	*Miniopterus*	*Miniopterus*	91.21%	F	4.6	A	Seq293_MF	MW684367

Supplementary File

Bat identifier	Bat species (morphological)	Bat species (molecular)	% id with reference	Sex	Forearm length	Age	Sequence identifier	Accession number
345	*Miniopterus*	*Miniopterus*	91.32%	F	4.66	A	Seq345_MF	MW684368
347	*Miniopterus*	*Miniopterus*	91.32%	F	4.76	A	Seq347_MF	MW684369
349	*Miniopterus*	*Miniopterus*	91.53%	M	4.55	A	Seq349_MF	MW684370
355	*Miniopterus*	*Miniopterus*	91.42%	F	4.65	A	Seq355_MF	MW684371
362	*Hipposideros lankadiva*	*Hipposideros*	95.42%	M	8.71	A	Seq362_HL	MW460890
374	*Hipposideros lankadiva*	*Hipposideros*	95.42%	F	8.78	A	Seq374_HL	MW460891
376	*Hipposideros lankadiva*	*Hipposideros*	95.42%	F	9.13	A	Seq376_HL	MW460892
378	*Hipposideros lankadiva*	*Hipposideros*	95.42%	F	8.76	A	Seq378_HL	MW460893

Abbreviations: M, Male; F, Female; A, Adult; SA, Sub-adult;

Supplementary File

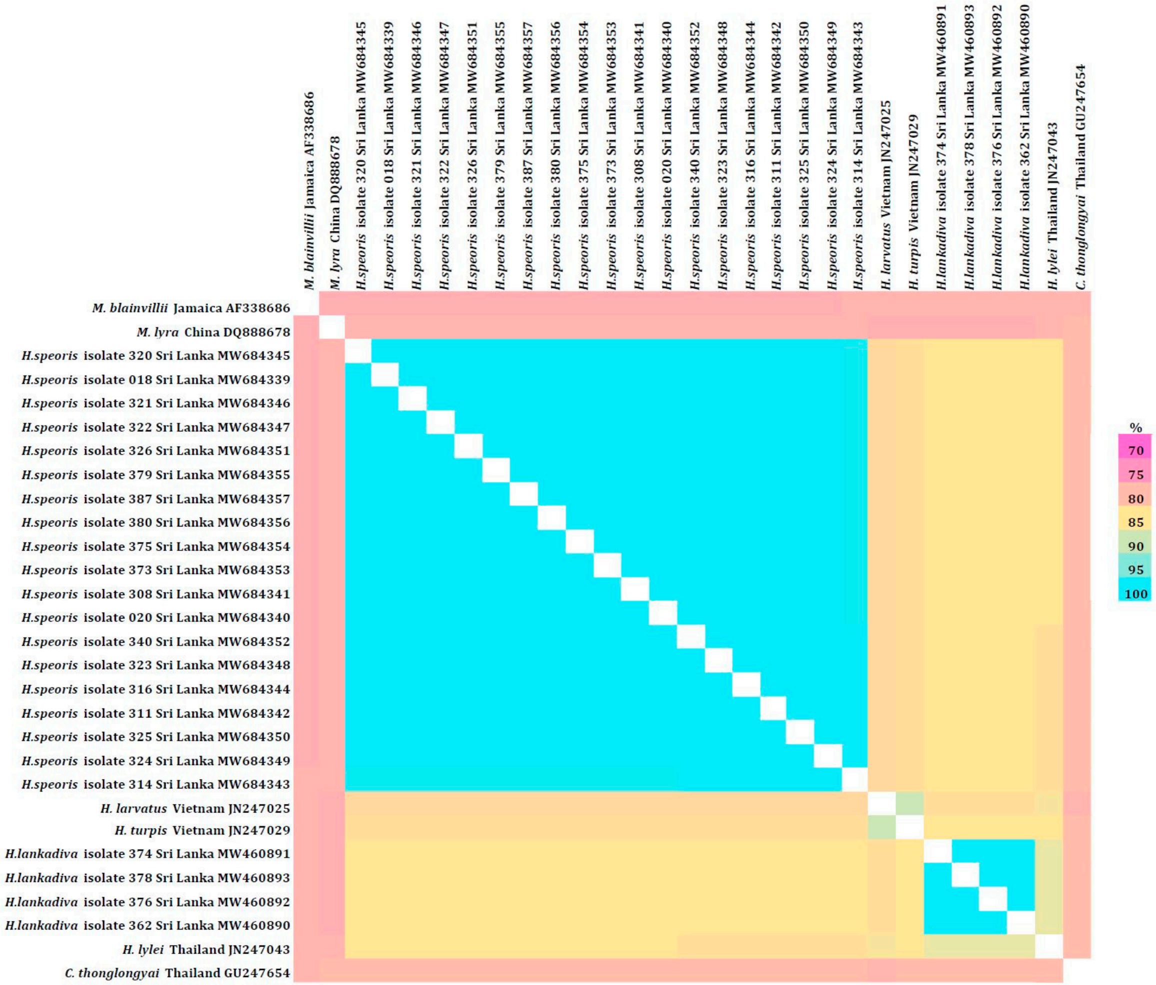

Figure S1. Heat maps based on the full MT-CYB gene (1,140 bp) of *Hipposideros speoris*. Percentage of identity is depicted by color ranging from 70 percent (red) to 100 percent (blue).

Supplementary File

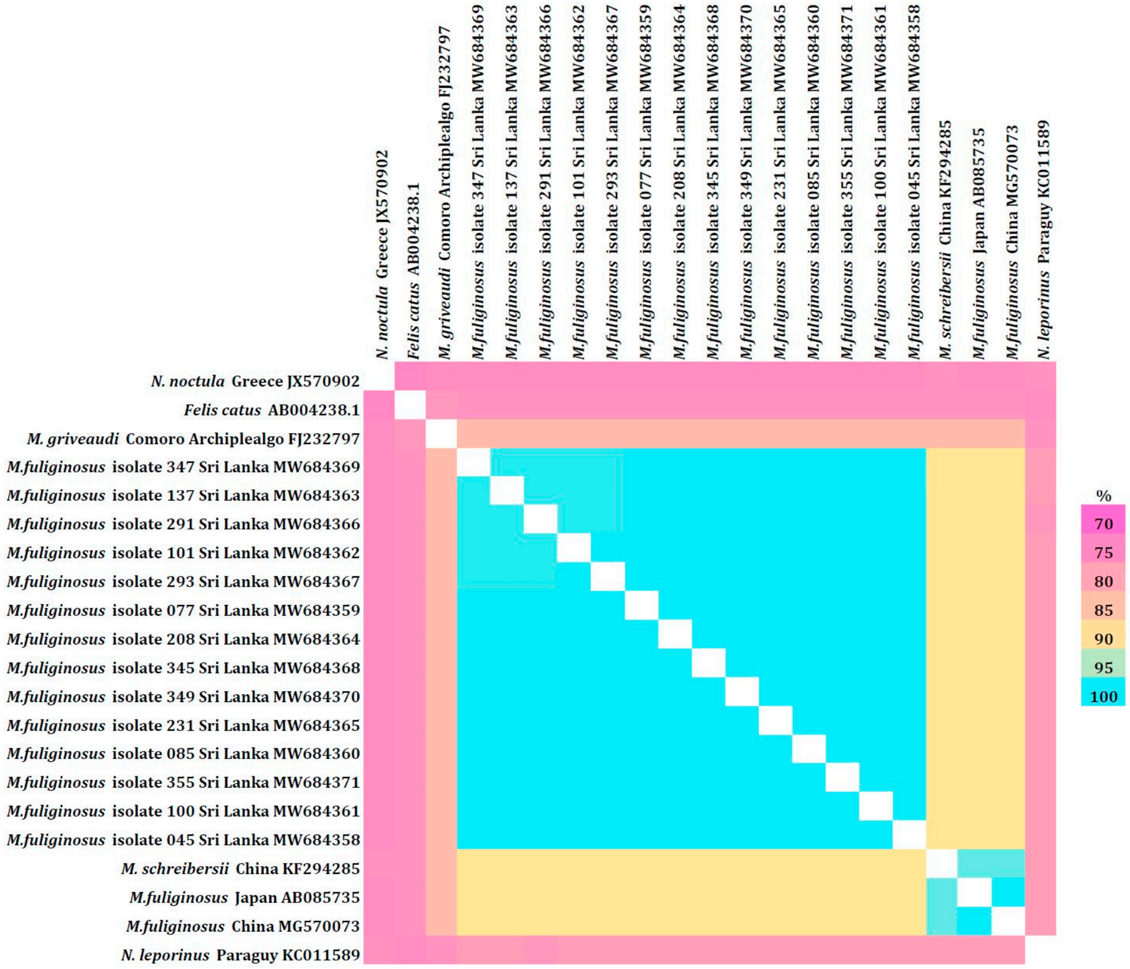

Figure S2. Heat maps based on the full MT-CYB gene (1,140 bp) of *Miniopterus fuliginosus*. Percentage of identity is depicted by color ranging from 70 percent (red) to 100 percent (blue).

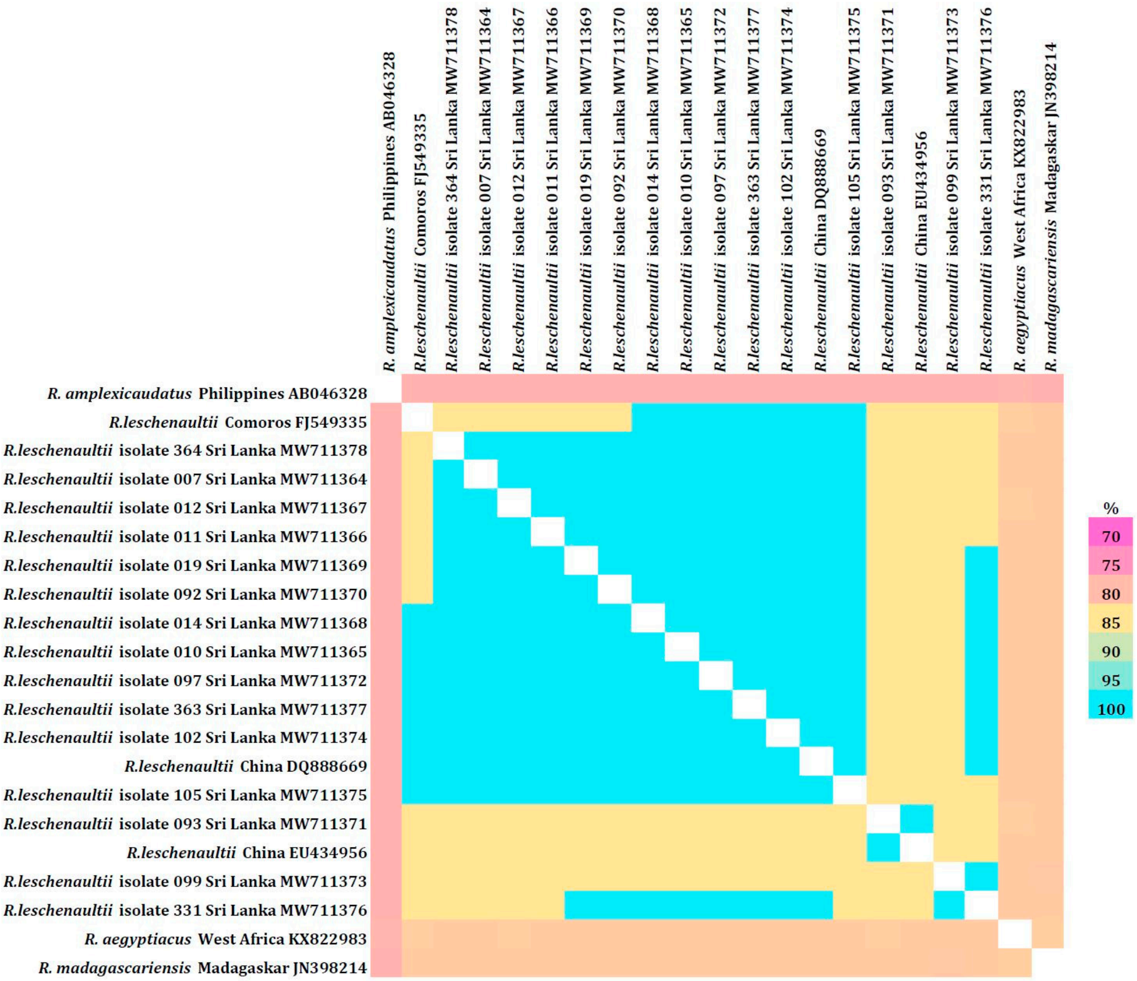

Figure S3. Heat maps based on the full MT-CYB gene (1,140 bp) of *Rousettus leschenaultii*. Percentage of identity is depicted by color ranging from 70 percent (red) to 100 percent (blue).

Supplementary File

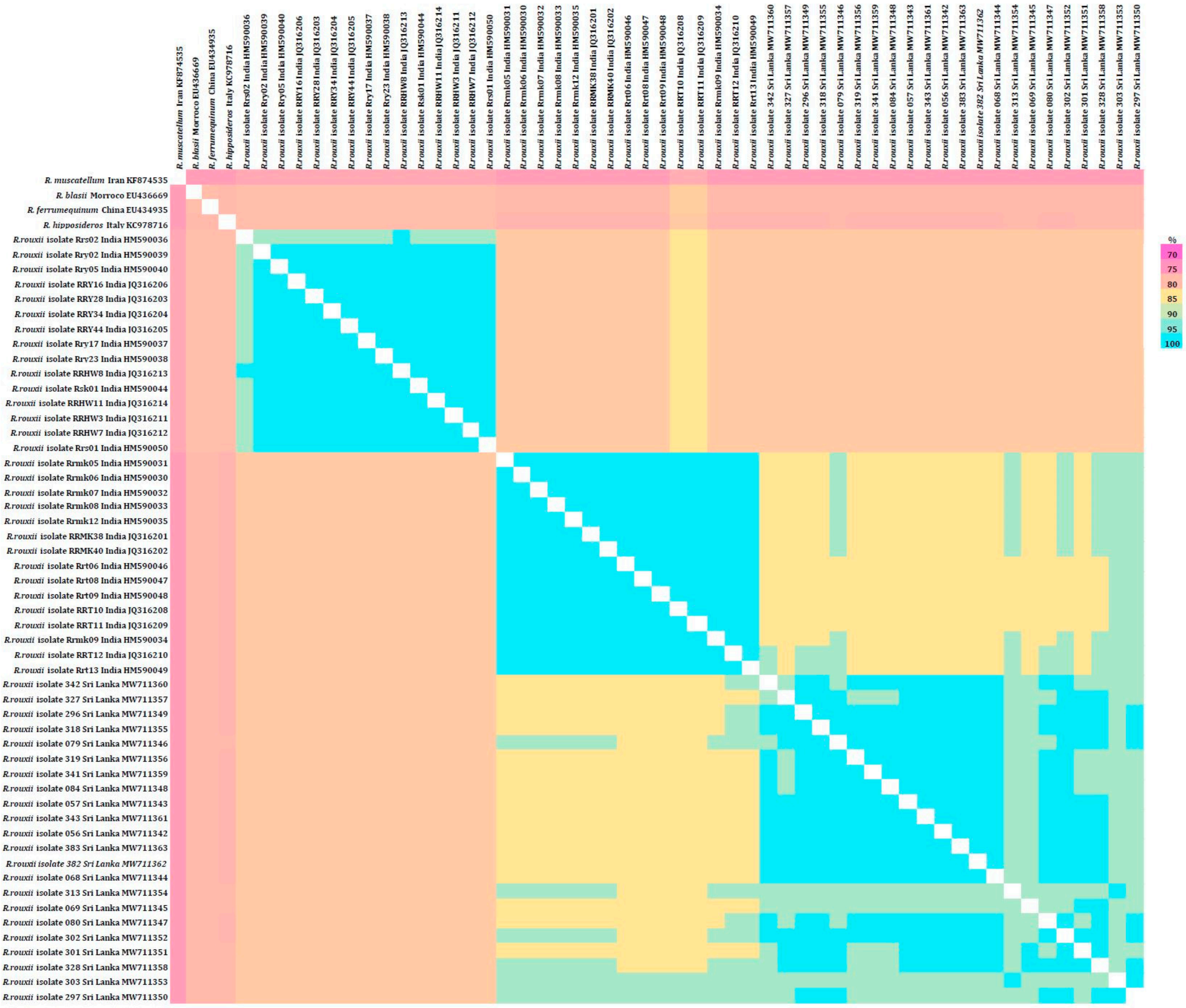

Figure S4. Heat maps based on the full MT-CYB gene (1,140 bp) of *Rhinolophus rouxii*. Percentage of identity is depicted by color ranging from 70 percent (red) to 100 percent (blue).
